# Hepatitis B Reactivation and Vaccination Effectiveness after Solid Organ Transplantation: A Matched Case-Control Study

**DOI:** 10.3390/vaccines12070804

**Published:** 2024-07-19

**Authors:** Yongseop Lee, Jaeeun Seong, Sangmin Ahn, Min Han, Jung Ah Lee, Jung Ho Kim, Jin Young Ahn, Nam Su Ku, Jun Yong Choi, Joon-Sup Yeom, Beom Kyung Kim, Su Jin Jeong

**Affiliations:** 1 Division of Infectious Diseases, Department of Internal Medicine, Yonsei University College of Medicine, Seoul 03722, Republic of Korea; yslee@yuhs.ac (Y.L.); sjaeeun1127@yuhs.ac (J.S.); cleverman7@yuhs.ac (S.A.); hmin0622@yuhs.ac (M.H.); peacefulee@yuhs.ac (J.A.L.); qetu1111@yuhs.ac (J.H.K.); comebacktosea@yuhs.ac (J.Y.A.); smileboy9@yuhs.ac (N.S.K.); seran@yuhs.ac (J.Y.C.); joonsup.yeom@yuhs.ac (J.-S.Y.); 2 Institute of Gastroenterology, Department of Internal Medicine, Yonsei University College of Medicine, Seoul 03722, Republic of Korea; beomkkim@yuhs.ac

**Keywords:** hepatitis B virus, vaccination, virus reactivation, transplantation, vaccine

## Abstract

Solid organ transplant (SOT) recipients are at significant risk of hepatitis B (HB) virus (HBV) reactivation (HBVr). Despite the clinical significance of HBVr after solid organ transplantation, data on the risk factors for HBVr and vaccine effectiveness in SOT recipients with resolved HBV infection are limited. This study evaluated the risk factors for HBVr and the seroconversion rates after HBV vaccination in SOT recipients. Patients who had undergone solid organ transplantation and those with a resolved HBV infection were identified. We matched patients who experienced post-transplantation HBVr with those who did not. We also explored the characteristics and seroconversion rates of HBV-vaccinated patients following transplantation. In total, 1299 SOT recipients were identified as having a resolved HBV infection at the time of transplantation. Thirty-nine patients experienced HBVr. Pre-transplant HB surface antibodies (anti-HBs) positivity and allograft rejection within 3 months after transplantation were independently associated with HBVr. Among the 17 HBV-vaccinated patients, 14 (82.4%) received three or fewer vaccine doses, and 13 (76.5%) had seroconversion with positive anti-HBs results. Pre-transplant anti-HBs(−) status and allograft rejection were risk factors for HBVr in SOT recipients with a resolved HBV infection, and HBV vaccination after transplantation resulted in a high rate of anti-HBs seroconversion. HBV vaccination after transplantation should be considered to reduce the HBVr risk.

## 1. Introduction

Hepatitis B (HB) virus (HBV) is a common chronic viral infection worldwide. It is estimated that one in every three people in the world has been exposed to HBV, and 296 million people have a chronic HBV infection [1,2,3]. HBV infection is especially common in the Western Pacific region, which includes South Korea and Africa, with a prevalence of 7.1% and 6.5%, respectively [4]. In addition, chronic HBV infection is a leading cause of liver cirrhosis and hepatocellular carcinoma, estimated to cause 820,000 deaths annually [1]. Although the introduction of HBV vaccination and hepatitis B immunoglobulin has greatly reduced the transmission of HBV, it remains a major global health problem [5].

HBV reactivation (HBVr) comprises an increase in viral replication in patients with chronic HBV infection or resolved HBV infection [6,7]. HBVr may be classified into two broad categories based on the baseline virologic profile: an increase in HBV DNA in patients who are positive for HB surface antigen (HBsAg) and the reappearance of HBsAg and HBV DNA in individuals who are initially negative for HBsAg and HBV DNA [8]. HBVr starts with viral replication, followed by liver injury that results from delayed immune reconstitution [7]. The severity of liver injury varies greatly among individuals, ranging from an asymptomatic increase in alanine transaminase levels to severe hepatitis or even liver failure [7].

Resolved HBV infection is associated with a substantially lower risk of HBVr compared to that associated with chronic HBV infection; however, this risk substantially increases with the administration of chemotherapy or immunosuppressants [6]. Resolved HBV infection indicates prior HBV exposure but self-limited infection [6]. These patients exhibit the disappearance of serum HBV DNA, the appearance of HB core antibodies (anti-HBc), and the seroconversion of HBsAg to HB surface antibodies (anti-HBs) during recovery [9]. Despite serologic resolution, traces of HBV DNA may persist in the hepatocytes in the form of covalently closed circular DNA (cccDNA) and integrated viral DNA [10,11,12]. Based on the presence of cccDNA, HBVr can occur with attenuation of immune regulation mediated by immunosuppression in the host. 

HBVr is also a major concern for solid organ transplant (SOT) recipients with HBV infection attributable to exposure to various immunosuppressants. The HBVr risk for HBsAg(+) kidney transplantation (KT) recipients is high at 45–70%; therefore, these patients should receive antiviral prophylaxis after transplantation [13,14,15,16]. In contrast, SOT recipients with resolved HBV infection face a lower, though not insignificant, risk of HBV reactivation, ranging from 4.7% to 6.5% [17,18,19,20], which is slightly lower than the 10% risk recommended by the guidelines for antiviral prophylaxis [21]. Although the risk of reactivation with resolved HBV infection is not as high as that with chronic HBV infection, it can be a substantial burden after transplantation in areas where HBV is highly endemic, and this risk can increase depending on the risk factors of individual patients. Therefore, it is necessary to further stratify the reactivation risk of SOT recipients to create an appropriate strategy to prevent reactivation in these patients.

However, data on the incidence of HBVr and the risk factors for HBVr in SOT recipients with resolved HBV infection are limited. The guidelines recommend either monitoring patients for reactivation or anti-HBV prophylaxis because there is no clear evidence indicating which approach is better [6]. In addition, no study has investigated the effectiveness of HBV vaccination in this population. Therefore, this study aimed to evaluate the risk factors for HBVr and the seroconversion rate after HBV vaccination in SOT recipients. 

## 2. Materials and Methods

### 2.1. Study Design and Study Population 

This matched case–control study was conducted at Severance Hospital, which is a large tertiary teaching hospital with 2400 beds in South Korea. We identified SOT recipients who underwent transplantation at this research hospital between January 2005 and April 2023. The procedures involved KT, liver transplantation (LT), lung transplantation (LuT), and heart transplantation (HT). The preoperative HBV serostatus was routinely assessed at the study institution based on anti-HBs, HBsAg, and anti-HBc. We excluded patients who did not undergo preoperative HBV serologic testing because the presence of HBV could not be determined. Patients with HBsAg(−) and anti-HBc(+) were defined as having resolved HBV infections and were included in this study. 

Cases of HBVr and controls were defined as instances of seroreversion from HBsAg(−) to HBsAg(+) after transplantation, and no instances of HBVr observed until the end of July 2023 (the data cutoff date), respectively. For each case, two controls were matched using propensity scores. Propensity scores were calculated using age, sex, transplanted organ, and year of transplantation as independent variables. 

To evaluate the efficacy of HBV vaccination in patients with resolved HBV infection, we identified individuals in our entire database who had received the HBV vaccine. We identified 39 patients who received the HBV vaccine and further reviewed 17 patients who were vaccinated post-transplant to determine the efficacy of HBV vaccination after transplantation. Detailed characteristics of each vaccine recipient were reviewed to identify individual immunocompromising factors and the risk of HBVr. Anti-HBs seroconversion rates were assessed to determine the efficacy of vaccination. 

### 2.2. Variables and Outcome Measures

The following variables were extracted using the institution’s data extraction system: demographic characteristics (age and sex), comorbidities, type of transplanted organ, pre-transplant desensitization, allograft rejection within the first 3 months post transplantation, and pre-transplant anti-HBs status. The Charlson comorbidity index was calculated to estimate the severity of comorbid conditions of SOT recipients [22]. Demographics and comorbidities were assessed based on the criteria used at the time of transplantation. The pre-transplant desensitization variable encompassed the administration of medications (anti-thymoglobulin, rituximab, and intravenous immunoglobulin) or plasmapheresis during the patient’s admission for transplantation. The pre-transplant anti-HBs status was determined using the most recent test results before transplantation. Allograft rejection was defined as a condition requiring high-dose glucocorticoid therapy for at least 120 h after transplantation, according to our institution’s transplantation protocol. 

Patients who received the HBV vaccine were identified by reviewing the vaccination records of the study hospital. To evaluate the efficacy of HBV vaccination after transplantation, patients who were vaccinated after transplantation were assessed. The following characteristics of recipients were collected: immunosuppressant use at the time of vaccination, medications used for desensitization and induction therapy, treatment of rejection prior to vaccination, total vaccine dose, follow-up duration, HBVr, and anti-HBs seroconversion. Anti-HBs seroconversion was defined as the confirmation of at least one instance of anti-HBs positivity (≥10 IU/L) after HBV vaccination. The variables were extracted with a cutoff date of the end of July 2023. 

### 2.3. Ethical Consideration 

The study was performed in compliance with relevant laws and institutional guidelines and the Institutional Review Board of the Severance Hospital approved this study (4-2023-1285). The requirement for patient consent was waived due to the retrospective nature and minimal risk of the study 

### 2.4. Statistical Analysis 

For the case–control matching, the propensity scores were calculated using the independent predictors of HBVr with a multivariable logistic regression model. SOT recipients with HBVr were matched on a 1:2 basis with patients without HBVr using individual propensity scores. Age, sex, transplanted organ, and year of transplantation were used as independent variables. 

Independent *t*-tests or Mann–Whitney U tests were used for continuous variables to compare clinical characteristics and outcomes. The Shapiro–Wilk test was performed to determine normality, and non-parametric tests were performed if the variables were not normally distributed. In contrast, Pearson’s chi-square tests or Fisher’s exact tests were used for categorical variables. Variables with a *p*-value < 0.05 from the univariable analysis were included in the multivariable logistic regression model to determine independent risk factors for HBVr. Use of pre-transplant immunoglobulin was excluded from the risk factor analysis according to the discretion of the investigators because the variable lacked biological plausibility for HBVr. A variance inflation factor >10 was used to indicate multicollinearity, and the Hosmer–Lemeshow test was performed to test goodness of fit. Survival analysis was conducted using the log-rank test to compare the risk of HBVr. Differences were considered statistically significant at *p* < 0.05. All statistical analyses were performed using R V.4.2.2 (The R Foundation for Statistical Computing, Vienna, Austria).

## 3. Results

### 3.1. Patient Characteristics 

**Figure 1:**
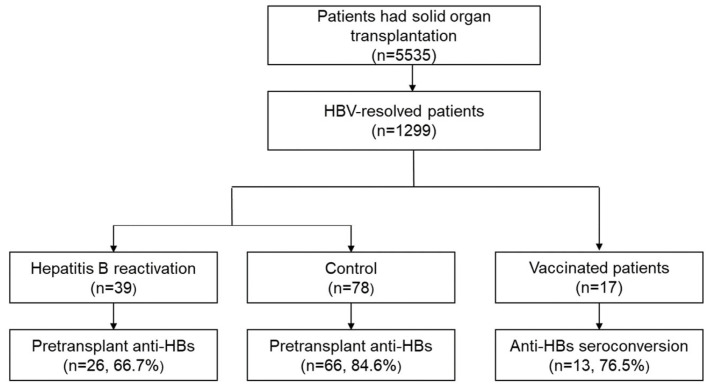
Flowchart depicting the selection of the study population. Anti-HBs, hepatitis B surface antibodies; HBV, hepatitis B virus.

In total, 1299 SOT recipients were identified as having resolved HBV infections at the time of transplantation. Among them, 1064 (81.9%) had pre-transplant anti-HBs, and antiviral prophylaxis was used for seven SOT recipients, none of whom experienced HBVr. The HBVr rates for the KT, LT, LuT, and HT groups were 2.88% (26/902), 2.55% (7/275), 6.25% (5/80), and 2.38% (1/42), respectively. A total of 39 patients experienced HBVr after transplantation and were matched to controls after propensity scores using age, sex, transplanted organ, and year of transplantation were calculated as independent variables. For each case, two controls with the nearest propensity score were matched. Therefore, 78 matched patients were included in the control group (Figure 1). 

The median age of the case group was 57 years (interquartile range [IQR]: 49–60 years), and that of the control group was 57 years (IQR: 50–61 years) (Table 1). The sex distribution did not differ significantly between the two groups (male patients: 74.4% in the case group vs. 69.2% in the control group; *p* = 0.565). Comorbidities including a history of transplantation and the Charlson comorbidity index score were not significantly different between the two groups. 

The most commonly transplanted organ was the kidney in both the case group (*n* = 26, 66.7%) and the control group (*n* = 57, 73.1%), followed by the liver (*n* = 7, 17.9%), lung (*n* = 5, 12.8%), and heart (*n* = 1, 2.6%) in the case group, and by the lung (*n* = 12, 15.4%), liver (*n* = 5, 6.4%), and heart (*n* = 4, 5.1%) in the control group. No significant differences in the types of transplanted organs were observed between the two groups. For pre-transplant desensitization therapy, intravenous immunoglobulin and rituximab were administered significantly more frequently to patients in the case group (17.9% and 25.6%, respectively) than to patients in the control group (5.1% and 10.3%, respectively). No significant difference in the use of anti-thymoglobin or plasmapheresis was observed between the two groups. The rate of allograft rejection within 3 months was significantly higher in the case group (30.8% in the case group vs. 14.1% in the control group; *p* = 0.032), and the rate of pre-transplant anti-HBs positivity was significantly higher in the control group (66.7% in the case group vs. 84.6% in the control group; *p* = 0.026).

### 3.2. Outcomes of Patients with HBVr 

The clinical outcomes of the 39 patients who experienced HBVr were evaluated (Table 2). 

Antiviral therapy was initiated in 82.1% (32/39) of patients, and of the seven patients who did not start antiviral therapy, one patient had negative HBsAg seroconversion without treatment. Liver cirrhosis occurred in 12.8% (5/39) of patients, including two KT and LuT recipients each and one LT recipient. No patients developed hepatocellular carcinoma or underwent additional LT, and mortality occurred in 20.5% (8/39) of the patients. One KT recipient died from acute liver failure due to HBVr.

### 3.3. Risk Factors for HBVr 

Binary logistic regression was used to analyze possible risk factors for HBVr (Table 3). 

The univariable analysis revealed that pre-transplant rituximab use (odds ratio [OR], 3.02; 95% confidence interval [CI], 1.09–8.66; *p* = 0.035) and allograft rejection within 3 months after transplantation (OR, 2.71; 95% CI, 1.06–6.98; *p* = 0.036) were associated with HBVr, whereas pre-transplant anti-HBs (OR, 0.36; 95% CI, 0.14–0.90; *p* = 0.029) was inversely associated with HBVr. In the multivariable analysis, pre-transplant anti-HBs (OR, 0.32; 95% CI, 0.12–0.82; *p* = 0.019) and allograft rejection within 3 months after transplantation (OR, 2.76; 95% CI, 1.02–7.53; *p* = 0.045) were independently associated with HBVr. Administration of rituximab also tended to increase the risk of HBVr, but it did not reach statistical significance. 

### 3.4. Time to HBVr According to Pre-Transplant Anti-HBs Status 

**Figure 2:**
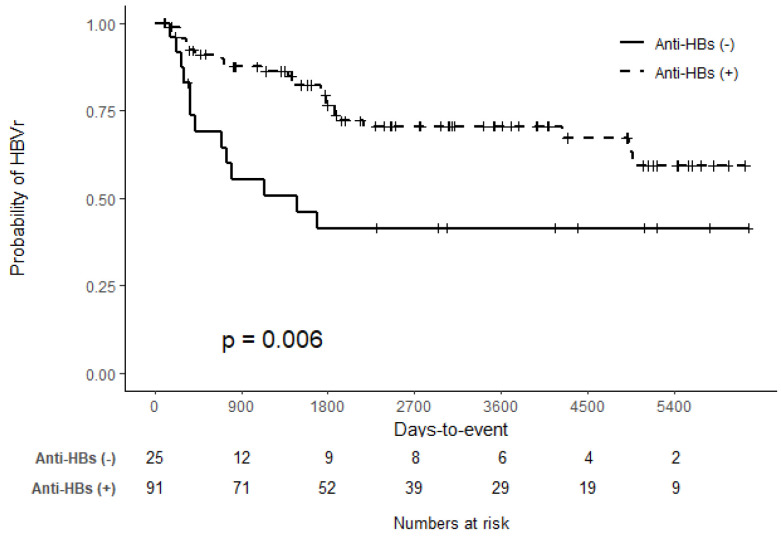
Kaplan–Meier curves of hepatitis B reactivation according to pre-transplant hepatitis B surface antibodies (anti-HBs) status.

Differences in the time to HBVr after SOT were analyzed according to the pre-transplant anti-HBs status. The Kaplan–Meier curves showed that pre-transplant anti-HBs(−) status was associated with HBVr (Figure 2). The median number of days to HBVr was 1411 (IQR: 360–1867) in patients positive for anti-HBs pre transplant, which was longer than the 417 (IQR: 308–804) days in patents negative for anti-HBs. However, the difference was not statistically significant (Table 4). 

However, HBVr after 1–3 years of transplantation was significantly higher in patients negative for anti-HBs, and the HBVr after 3 years was higher in patients positive for anti-HBs. HBVr within 1 year after transplantation did not differ between the groups.

### 3.5. Efficacy of HBV Vaccination in Pateints with Resolved HBV after Solid Organ Transplantation

HBV vaccination was administered to 17 patients with resolved HBV after transplantation. The mean age of the patients was 48.6 years (standard deviation: 18.7 years), including two children at the time of transplantation. KT and LT were the most common, each performed in seven patients (41.2%). One patient underwent HT and two patients underwent LuT. All recipients received steroid pulse therapy for transplant induction therapy, and one recipient received additional rituximab and plasmapheresis for desensitization. None of HBV-vaccinated recipients experienced rejection requiring treatment prior to vaccination. 

Vaccination was administered once to five (29.4%) patients, three times to nine patients (52.9%), and more than three times to three patients (17.7%). HBV vaccination started in 52.9% of patients in the first year after transplant, 23.5% in the second year, and in the subsequent period for the remaining 23.5%. Patients initially received vaccinations at least 6 months apart from the date of transplant. However, there was one patient who was vaccinated on post-transplant day 2. A total of 13 patients exhibited anti-HBs-positive seroconversions (76.5%). None of patients who responded to HBV vaccination experienced HBVr; however, one patient who did not respond to HBV vaccination experienced HBVr. Detailed information on vaccinated patients is presented in Supplementary Table S1. 

## 4. Discussion 

The present study aimed to evaluate the risk factors for HBVr and vaccination effectiveness in HBsAg(−) and anti-HBc(+) patients after SOT. HBVr was observed in 3.0% of the study population, and pre-transplant anti-HBs status and allograft rejection were identified as risk factors for HBVr. Seventeen patients received HBV vaccination post-transplant, and 76.5% of them exhibited seroconversion from anti-HBs(−) to anti-HBs(+). 

The strength of this study lies in its presentation of real-world data on the reactivation risk in SOT recipients with resolved HBV infection. While the risk of HBVr in anti-HBc(+) patients has been extensively investigated in patients with hematological malignancies and in LT recipients, limited real-world information is available for KT or LuT recipients [23]. Yin et al. conducted a meta-analysis and reported a 2.5% incidence rate of HBVr in non-liver SOT recipients with resolved HBV infection [24]. However, among the 1sixteen6 included studies, thirteen were conducted including KT recipients, and only one included LuT recipients. Only a single study has examined the development of HBVr in LuT recipients, reporting HBVr in 1 of 11 anti-HBc(+) recipients [23]. The current study included 80 LuT recipients with anti-HBc(+). Among them, 6.25% developed HBVr, which was higher than that in the other SOTs. 

In addition, this study included a large number of SOT recipients with resolved HBV infections. HBV infection rates are highest in the WHO Western Pacific Region, which includes South Korea [25]. In this study, 23.5% of SOT recipients were HBV-resolved patients, which is a substantial proportion. Resolved HBV infection is relatively infrequent in the Western population, thus prophylaxis or vaccination of these patients during SOT has not been adequately studied [26]. However, the results of this study suggest that SOT is frequently performed in patients with resolved HBV infection in high-endemic areas. Therefore, it is necessary to develop effective prevention strategies for HBVr in these populations. 

This study identified anti-HBs negativity and graft rejection as independent risk factors for HBVr in SOT recipients with resolved HBV infection. The role of anti-HBs in resolved HBV infection has been well-studied in patients with hematological malignancies. A meta-analysis published in 2017 showed that anti-HBs(+) status reduced the risk of HBVr and had a significant protective effect with an OR of 0.21 [27]. The protective effect of anti-HBs against HBVr has been observed in several studies involving KT recipients [16,19,28,29]. Similar to previous studies, our study also demonstrated that anti-HBs has a protective effect on HBVr. In addition, Meng et al. demonstrated that graft rejection significantly increased the risk of HBVr [30]. Our study also showed that allograft rejection requiring high-dose glucocorticoid therapy was an independent risk factor for HBVr with an OR of 2.76. Based on these findings, more intensive HBV prophylaxis strategies are needed for anti-HBs(−) patients and those with allograft rejection that requires high-intensity immunosuppressive therapy. 

The development of HBVr can cause significant liver-related morbidity and mortality after SOT. The frequency of liver-related morbidity varies across studies. A meta-analysis of non-liver SOT recipients included eight studies that reported liver-related outcomes of HBVr [24]. Liver cirrhosis occurred in 18.9% of the patients, ranging from 0% to 62.5% across studies. HBV-related death occurred in 11.0% of the patients, with rates ranging from 0% to 50%. In our study, although a large proportion of patients initiated antiviral therapy, 12.8% developed post-transplant liver cirrhosis. One patient developed hepatic failure owing to HBVr, which resulted in death. Although these results are lower than those of the previous meta-analysis, the heterogeneity of the studies included in the meta-analysis makes making direct comparisons challenging. In addition, the independent contribution of HBVr was difficult to assess in organ transplant recipients because the concomitant use of multiple agents with hepatic toxicity is common.

This study is important because it evaluated the effectiveness of HBV vaccination in SOT recipients with resolved HBV infections. In the general population, HBV vaccination is more than 90% effective, but the response to the HBV vaccine is reduced in immunocompromised patients [31,32]. Therefore, the Infectious Diseases Society of America guidelines for the vaccination of an immunocompromised host recommend that the HBV vaccine be administered to anti-HBs(−) patients, preferably in the pre-transplant period in SOT candidates. Post-transplant vaccination is advised only if pre-transplant vaccination was not accomplished [33]. However, the effectiveness of post-transplant vaccination has not been sufficiently evaluated, especially in cases of resolved HBV infection. In this study, 235 recipients were identified as anti-HBs(−) before transplantation, but only 22 of them were vaccinated pre-transplant. Even including the 17 vaccinated recipients post-transplant, we found that HBV vaccination is performed in a small proportion of transplant recipients with resolved HBV infection. However, we observed that the seroconversion rate after vaccination was as high as 73%, which may be an important basis for recommending HBV vaccination after SOT. Based on the protective effects of anti-HBs against HBVr and the high seroconversion rate of vaccination, HBV vaccination after SOT should be considered. 

The effectiveness of HBV vaccination after SOT in reducing the risk of HBVr has not been studied. However, several studies focusing on the effectiveness of the HBV vaccine after hematopoietic stem cell transplant suggest promising outcomes [34,35,36,37]. Onozawa et al. reported that 39% of unvaccinated patients experienced HBVr after an allogeneic hematopoietic stem cell transplant, compared to none of the vaccinated patients [35]. Hammond et al. also reported that HBV vaccination after allogeneic hematopoietic stem cell transplant can significantly reduce the risk of HBVr in patients with resolved HBV infection [37]. These findings provide additional support for HBV vaccination in patients after SOT, with the expectation that it will also reduce the long-term HBVr risk. 

This study had several limitations. First, this was a single-center, retrospective study. Selection and information biases may have occurred due to the innate nature of the study design. Furthermore, this study included a small sample size, which may not have been statistically significant. Although we found a high HBVr rate in patients with LuT, reaching a meaningful conclusion was challenging because of the small sample size and limitations of the study design. 

In addition, although each SOT has unique characteristics, we included patients with various SOTs because of the small sample size. The heterogeneity of the study population requires caution when interpreting the results. Donor anti-HBc and HBsAg status has not been investigated in this study and can also be a confounding factor. In addition, despite the high seropositive conversion rate following HBV vaccination, we could not evaluate the reduction in HBVr risk after vaccination. Therefore, a multicenter prospective cohort study would be helpful to accurately assess the incidence of HBVr and its risk factors in SOT recipients with resolved HBV infection, especially LuT recipients. Furthermore, a randomized controlled trial is required to evaluate the effectiveness of vaccination in reducing HBVr risk.

In conclusion, we presented that a notable proportion of SOT recipients had resolved HBV infections; however, only a small proportion received HBV vaccination or antiviral prophylaxis. There is need to develop an effective preventive strategy for HBVr in SOT recipients with resolved HBV infection, especially in highly endemic areas. We found that graft rejection and anti-HBs negativity are risk factors for HBVr; therefore, more intensive HBV prophylaxis strategies are necessary for patients with these risk factors. We also found that HBV vaccination after SOT resulted in a high rate of anti-HBs seroconversion. Based on these findings, HBV vaccination after SOT should be considered to effectively prevent HBVr. Further studies are needed to evaluate the effectiveness of vaccination in reducing HBVr and optimize preventive strategies between HBV vaccination and antiviral prophylaxis after SOT. 

## Figures and Tables

**Table 1:** Comparison of patients with reactivated Hepatitis B and matched controls.

	Number (%)		
	Control (*n* = 78)	HBVr (*n* = 39)	*p*-Value
Age (years)	57.0 (50.0–61.0)	57.0 (49.0–60.0)	0.683
Male sex	54 (69.2%)	29 (74.4%)	0.565
Comorbidities			
Transplantation history	4 (5.1%)	4 (10.3%)	0.300
Myocardial infarction	9 (11.5%)	3 (7.7%)	0.518
Diabetes mellitus	21 (26.9%)	8 (20.5%)	0.449
Liver disease	5 (6.4%)	5 (12.8%)	0.242
Renal disease	58 (74.4%)	28 (71.8%)	0.767
Solid tumor	1 (4.3%)	3 (13.0%)	0.295
Charlson comorbidity index	2.0 (2.0–4.0)	2.0 (2.0–4.0)	0.559
Transplanted organ			
Kidney transplantation	57 (73.1%)	26 (66.7%)	0.472
Liver transplantation	5 (6.4%)	7 (17.9%)	0.052
Lung transplantation	12 (15.4%)	5 (12.8%)	0.711
Heart transplantation	4 (5.1%)	1 (2.6%)	0.518
Living donor transplantation	49 (62.8%)	25 (64.1%)	0.892
Pre-transplant desensitization			
Anti-thymoglobulin	9 (11.5%)	6 (15.4%)	0.557
Intravenous immunoglobulin	4 (5.1%)	7 (17.9%)	0.025
Rituximab	8 (10.3%)	10 (25.6%)	0.030
Plasmapheresis	8 (10.3%)	8 (20.5%)	0.128
Allograft rejection within 3 months	11 (14.1%)	12 (30.8%)	0.032
Pre-transplant anti-HBs	66 (84.6%)	26 (66.7%)	0.026

Anti-HBs, hepatitis B surface antibodies; HBVr, hepatitis B virus reactivation.

**Table 2:** Outcomes of hepatitis B reactivation after solid organ transplantation.

	Hepatitis B Reactivation (*n* = 39)
HBeAg(+)	20/27 (74.1%)
Anti-HBe	6/24 (25.0%)
Antiviral therapy initiation	32 (82.1%)
Liver cirrhosis	5 (12.8%)
Hepatocellular carcinoma	0 (0%)
Liver transplantation	0 (0%)
Mortality	8 (20.5%)

Anti-HBe, hepatitis B envelope antibodies; HBeAg, hepatitis B envelope antigen.

**Table 3:** Risk factors of hepatitis B reactivation after solid organ transplantation.

	Univariable Analysis		Multivariable Analysis	
	OR (95% CI)	*p*-Value	OR (95% CI)	*p*-Value
Rituximab	3.02 (1.09–8.66)	0.035	2.83 (0.95–8.59)	0.060
Allograft rejection	2.71 (1.06–6.98)	0.036	2.76 (1.02–7.53)	0.045
Pre-transplant anti-HBs	0.36 (0.14–0.90)	0.029	0.32 (0.12–0.82)	0.019

Anti-HBs, hepatitis B surface antibodies; OR, odds ratio; CI, confidence interval. Allograft rejection was included if it occurred within 3 months of transplantation.

**Table 4:** Time to hepatitis B reactivation stratified with pre-transplant anti-HBs status.

	Number (%)		
	Anti-HBs(+)	Anti-HBs(−)	*p*-Value
<1 year	7 (26.9%)	4 (30.8%)	0.801
1–3 years	4 (15.4%)	6 (46.2%)	0.038
>3 years	15 (57.7%)	3 (23.1%)	0.041
Median days to HBVr	1411 (360–1867)	417 (308–804)	0.054

Anti-HBs, hepatitis B surface antibodies; HBVr, hepatitis B virus reactivation.

## Data Availability

The datasets used for the current study are available from the corresponding author upon reasonable request.

## Supplementary Materials

The following supporting information can be downloaded at: https://www.mdpi.com/article/10.3390/vaccines12070804/s1, Table S1. Detailed characteristics of vaccinated patients.
