# Supplementary material for: Hepatitis B Reactivation and Vaccination Effectiveness after Solid Organ Transplantation: A Matched Case-Control Study
Source: Vaccines (Basel). 2024 Jul 19;12(7):804. doi: 10.3390/vaccines12070804 (PMC11281428; doi:10.3390/vaccines12070804)
Supplement: Supplementary file 1 [file vaccines-12-00804-s001.zip › vaccines-3085065-supplementary.docx]

**Supplementary** **Table S1. Detailed characteristics of vaccinated patients.**

| **Case** | **Sex/Age** | **Transplant year** | **Transplanted organ** | **Total dose** | **Immunosuppressant (mg)** | **Anti-HBs(+) seroconversion** | **HBVr** | **FU years** |
| --- | --- | --- | --- | --- | --- | --- | --- | --- |
| 1 | M/62 | 2006 | Kidney | 1 | MP (250), CsA, MPS | + | - | 12.2 |
| 2 | F/43 | 2010 | Kidney | 3 | DFZ (6), CsA, MPS | + | - | 13.3 |
| 3 | M/0 | 2011 | Liver | 3 | DFZ (3), Tac | - | + | 5.2 |
| 4 | M/55 | 2012 | Kidney | 3 | Tac, MMF | + | - | 11.1 |
| 5 | M/48 | 2012 | Kidney | 1 | PL (5), Tac, MPS | + | - | 11.1 |
| 6 | F/63 | 2013 | Kidney | 3 | DFZ (12), Tac, MMF | + | - | 10.2 |
| 7 | F/51 | 2014 | Kidney | 1 | PL (10), Tac, MMF | + | - | 8.8 |
| 8 | M/7 | 2014 | Liver | 1 | PL (2.5), Tac | + | - | 9.3 |
| 9 | M/59 | 2015 | Liver | 3 | PL (10), Tac, MMF | - | - | 8.0 |
| 10 | F/36 | 2016 | Kidney | 1 | PL (5), Tac, MMF | + | - | 6.8 |
| 11 | M/57 | 2017 | Heart | 5 | Tac, MMF | + | - | 5.8 |
| 12 | F/56 | 2019 | Liver | 3 | DFZ (6), Tac, everolimus | + | - | 3.8 |
| 13 | M/47 | 2021 | Lung | 6 | PL (10), Tac, MMF | - | - | 2.0 |
| 14 | M/58 | 2021 | Lung | 6 | PL (10), Tac, MMF | - | - | 2.0 |
| 15 | M/57 | 2021 | Liver | 3 | DFZ (12), Tac, MMF | + | - | 2.3 |
| 16 | M/68 | 2022 | Liver | 3 | DFZ (6), Tac, everolimus | + | - | 1.0 |
| 17 | M/60 | 2022 | Liver | 3 | Tac, MMF | + | - | 1.2 |

Immunosuppressant type and dose were investigated at the time of vaccination. In the case of HBVr, the FU years was censored and no deaths occurred. All recipients received steroid pulse therapy for transplant induction, and the 16th patient received additional rituximab and plasmapheresis.

HBVr, Hepatitis B virus reactivation; FU, follow up; M, male; F, female; MP, methylprednisolone; CsA, cyclosporine; MPS, mycophenolate sodium; Deflazacort, DFZ; Tac, tacrolimus; MMF, mycophenolate mofetil; PL, prednisolone.
